# Non-invasive and non-destructive measurements of confluence in cultured adherent cell lines

**DOI:** 10.1016/j.mex.2014.11.002

**Published:** 2014-11-25

**Authors:** Steven Busschots, Sharon O’Toole, John J. O’Leary, Britta Stordal

**Affiliations:** a Department of Histopathology, Trinity College Dublin, Central Pathology Laboratory, St. James's Hospital, Dublin 8, Ireland; b Department of Obstetrics and Gynaecology, Trinity College Dublin, Trinity Centre, St James's Hospital, Dublin 8, Ireland; c Department of Natural Sciences, Hendon Campus, Middlesex University, London NW4 4BT, United Kingdom

**Keywords:** Area Fraction Output Attached Cell Confluency, Confluence, Cell count, Phase-contrast microscopy, ImageJ, Area fraction, Non-destructive

## Abstract

Many protocols used for measuring the growth of adherent monolayer cells *in vitro* are invasive, destructive and do not allow for the continued, undisturbed growth of cells within flasks. Protocols often use indirect methods for measuring proliferation. Microscopy techniques can analyse cell proliferation in a non-invasive or non-destructive manner but often use expensive equipment and software algorithms. In this method images of cells within flasks are captured by photographing under a standard inverted phase contract light microscope using a digital camera with a camera lens adaptor. Images are analysed for confluence using ImageJ freeware resulting in a measure of confluence known as an Area Fraction (AF) output. An example of the AF method in use on OVCAR8 and UPN251 cell lines is included.

• Measurements of confluence from growing adherent cell lines in cell culture flasks is obtained in a non-invasive, non-destructive, label-free manner.
• The technique is quick, affordable and eliminates sample manipulation.
• The technique provides an objective, consistent measure of when cells reach confluence and is highly correlated to manual counting with a haemocytometer. The average correlation co-efficient from a Spearman correlation (*n* = 3) was 0.99 ± 0.008 for OVCAR8 (*p* = 0.01) and 0.99 ± 0.01 for UPN251 (*p* = 0.01) cell lines.

## Graphical Abstract

**Figure:**
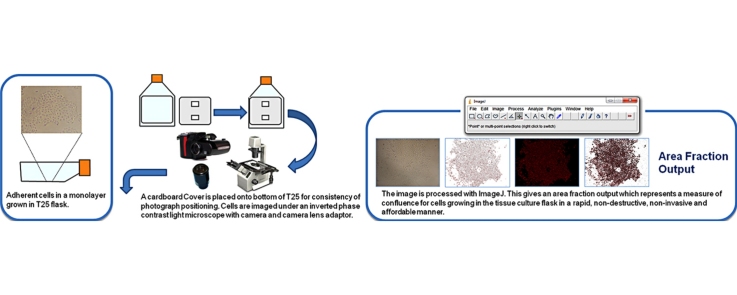

## Method

This is a novel, non-invasive and non-destructive method developed to calculate a measure of confluence for growing adherent cells without disturbing growth. The result is known as an area fraction (AF) output which represents the amount of surface area that cells cover within a photographed area of a flask, representing an approximate value of confluence for the flask as a whole. This method can be used in order to monitor cells growing in T25 flasks or other cell culture plastics to determine objectively when they have reached confluence. This method can also be adapted for T75 flasks.

## Recommended equipment

• ImageJ 1.45 s [1] (http://imagej.nih.gov/ij/download.html).
• Inverted Nikon TMS Phase Contrast Microscope (Nikon, Surrey, United Kindgom).
• Nikon Coolpix camera lens adapter (eBay Inc., US, seller-imaging_apparatus, United States).
• Nikon Coolpix 4500 camera (Nikon, Surrey, United Kingdom).
• Tissue Culture Flask T25, with red vented cap (Sarstedt, Wexford, Ireland).

**Fig. 1:**
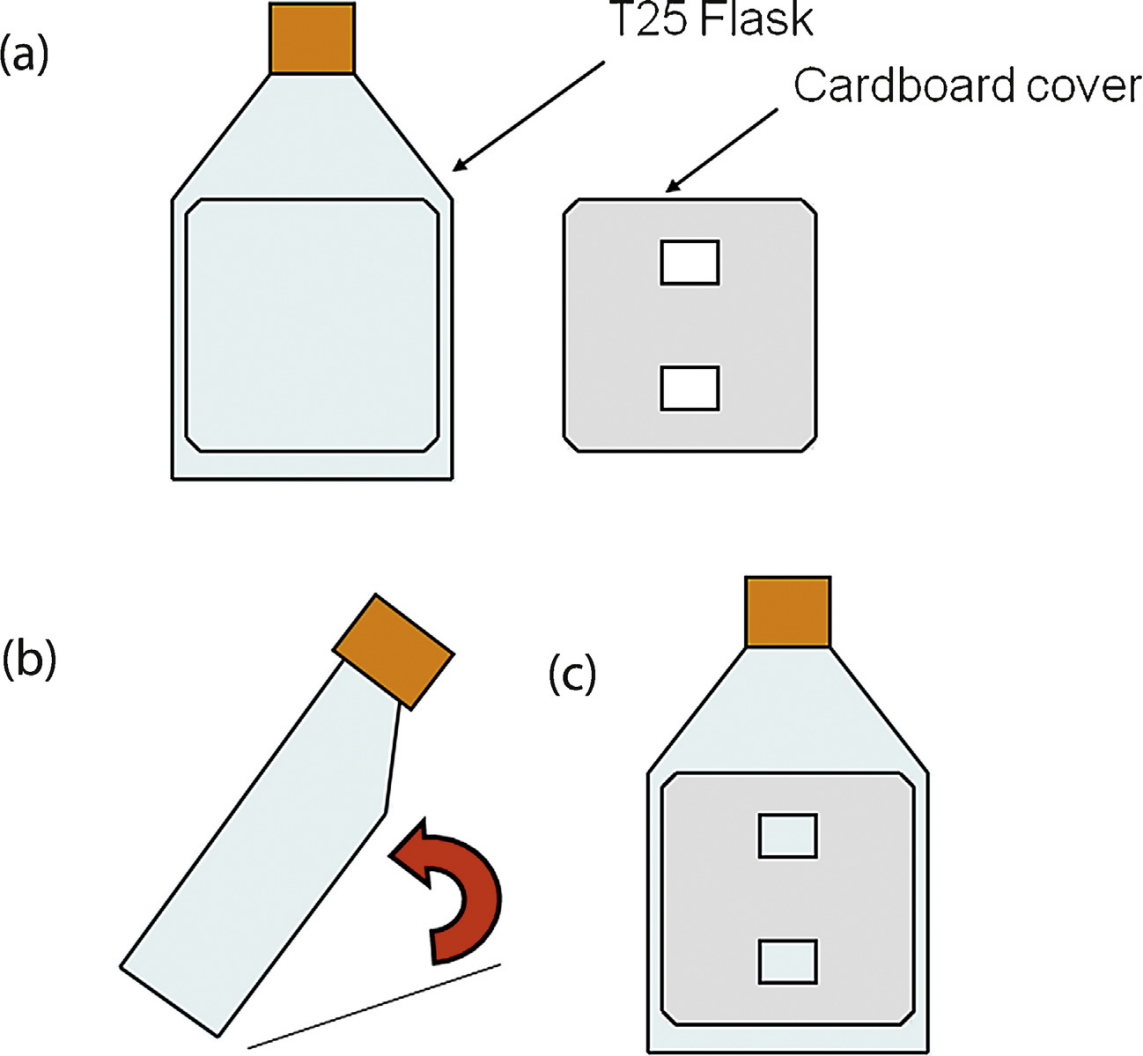
T25 cardboard cover design for AF calculation method. (a) Shows the cardboard cover design next to T25 flask. (b) and (c) show how cover is placed onto a T25 flask before photography.

Comparable products can be used for Microscope, camera, camera attachments and cell culture plastics. The cardboard cover slip used in this method (Fig. 1) is fashioned by measuring the flask dimensions and cutting cardboard into the correct shape using scissors.

## Microscope preparation and photographing

**Fig. 2:**
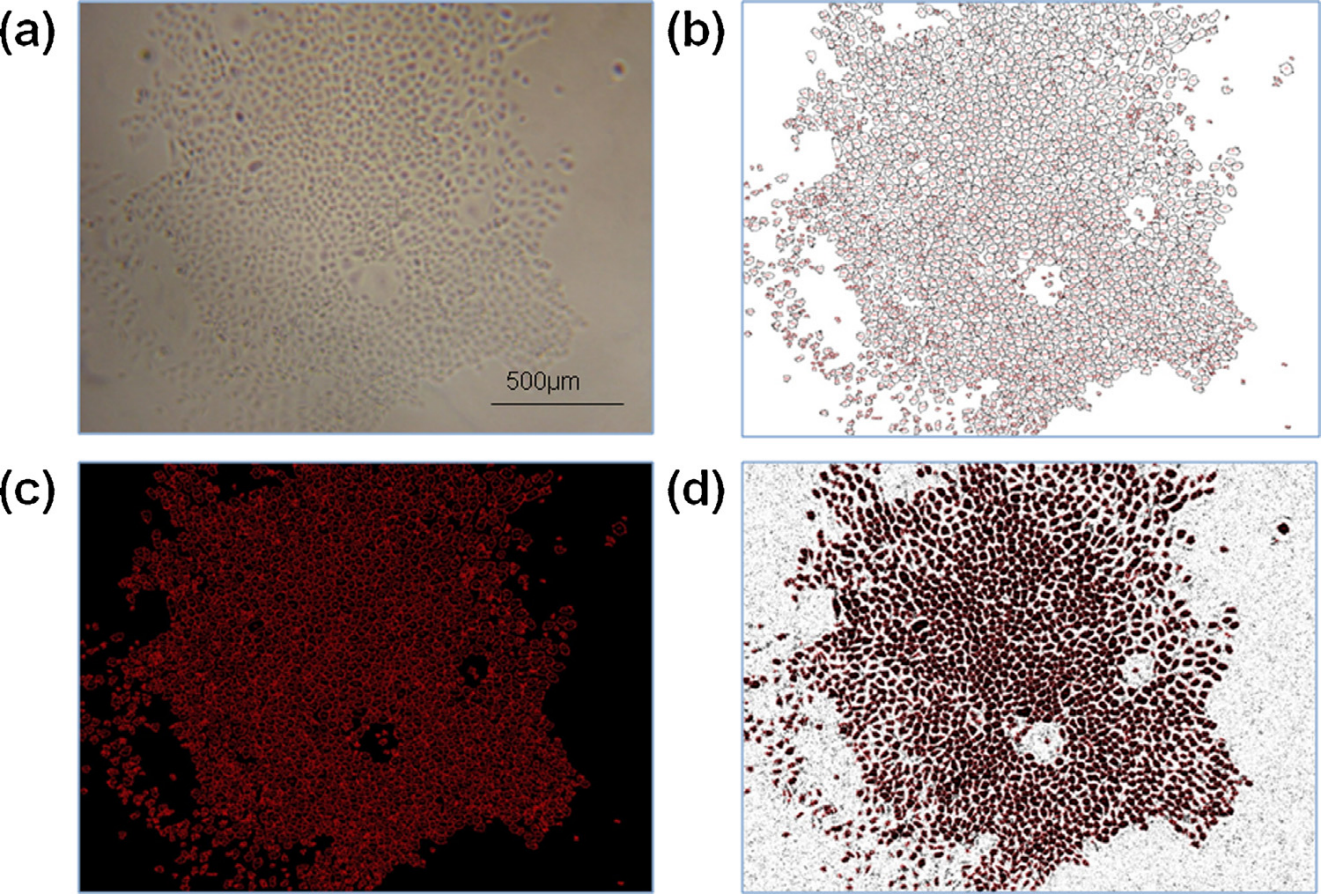
Example of images generated from the AF calculation method. (a) Original image generated from section 2.3.1 of UPN251 cells. (b) Image ‘Drawing of X.JPG’ generated after analysis step 7, represents the cells counted from the analysis of original image. (c) Image ‘Drawing of X.JPG’ after accuracy step 5. This is the image that is used in the overlay onto the original image, to check for accuracy of the generated count versus the cells present in original image. (d) The result of the merged images (‘a’ and ‘c’) after completion of accuracy steps. In this case the count looks accurate as each cell is labelled with red numbering.

**Fig. 3:**
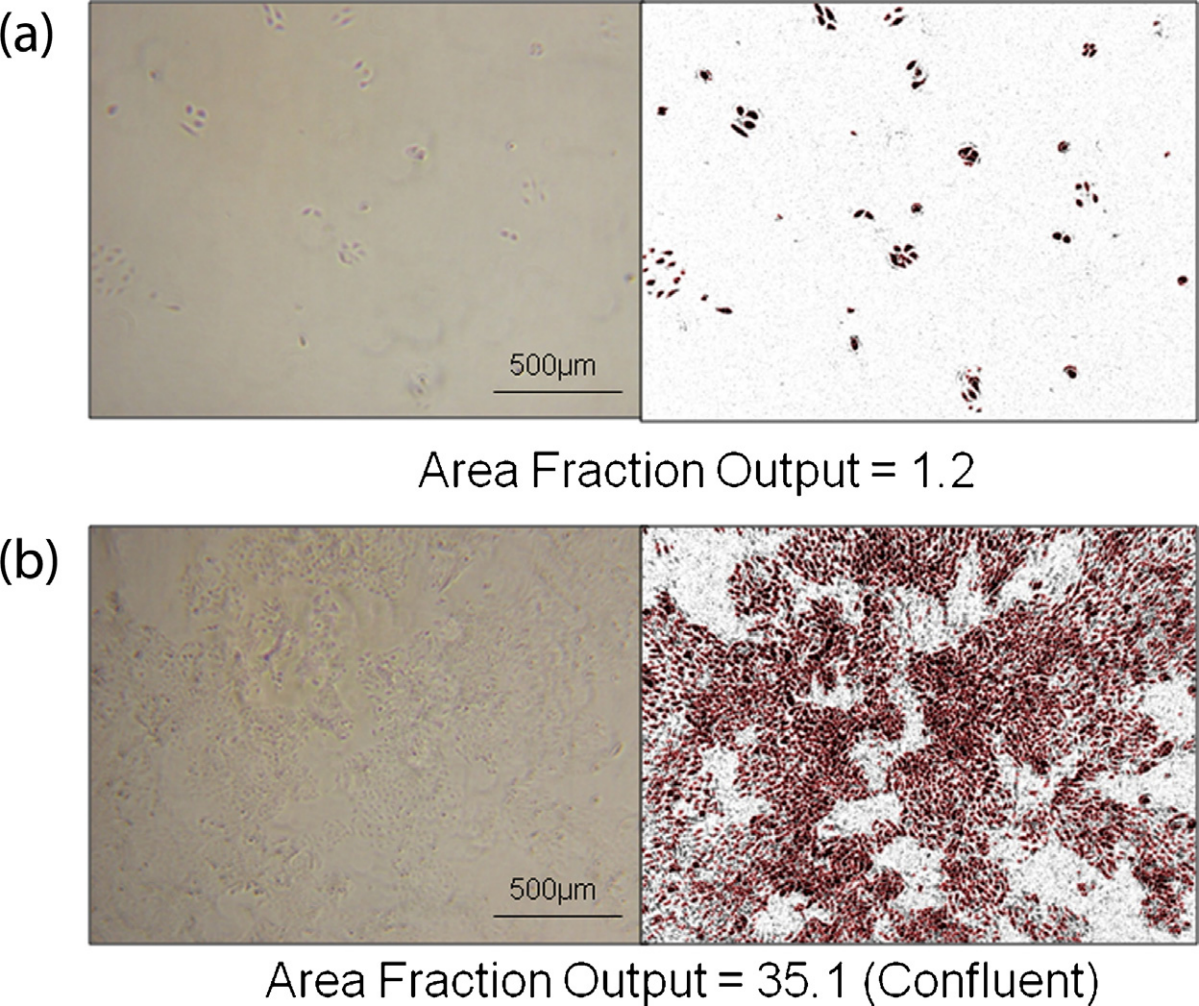
AF output photo analysis of newly seeded versus confluent flasks. The left image is the original phase contrast photograph; the right is after analysis with ImageJ software. (a) OVCAR8 cells after seeding. AF value of 1.2 was achieved in this case. (b) OVCAR8 upon reaching confluence. AF value of 35.1 was achieved in this case.

1. The inverted Nikon (TMS) phase contrast light microscope is focused with the 4× objective lens in place.
2. For use with the Nikon Coolpix camera the Nikon Coolpix camera lens adapter is screwed onto the camera lens of the Nikon Coolpix 4500 camera. One of the eyepieces of the microscope is removed and stored safely. The camera lens adapter with attached camera is placed into the occular tube of the microscope. (When using other camera and microscope systems the camera is set up as per the cameras user manual.)
3. The flask of cells are taken from the incubator. The cardboard cover slip, designed to fit the bottom of the flask having 1 cm holes close to the top and bottom of the flask, is placed onto the bottom of the flask. See Fig. 1.
4. The objective lens is placed over the hole in the cover slip and microscope is adjusted to focus on the cell nuclei to the sharpest on the LCD screen of the camera.
5. The Nikon Coolpix camera is zoomed in to a focus point of F4.0.
  a The microscope must be focused so that the cell nuclei are clearly visible on the camera’s LCD display. The cytoplasmic region of the cell will therefore be slightly out of focus. When using a different camera and microscope, the zoom settings may differ. In this case the camera must be zoomed in consistently so that cells appear as in Fig. 2(a) on the LCD display screen.
6. Two photographs are taken of the cells, one from the top and one from the bottom hole of the cardboard cover slip placed under the flask.
  a This ensures that photographs can be taken consistently in the same position of the flask over different time points for long term monitoring of confluence without disturbing growth.
  b If the cells grow evenly dispersed taking the photo anywhere within the 1 cm^2^ hole is sufficient for a consistent measure of confluence.
  c If the cells grow in clumps it is advised to track the largest colony of cells found within this 1 cm^2^ region.
7. At this point cells are placed back into the incubator. Analysis of photographs can be preformed at the researchers leisure.
  a Images generated by the Nikon Coolpix 4500 camera will be in JPEG format. ImageJ can process images in a variety of formats including, TIFF, GIF, JPEG, PNG, DICOM, BMP, PGM and FITS images. ImageJ also supports 8-bit, 16-bit and 32-bit (real) grayscale images and 8-bit and 32-bit colour images.
  Image analysis
8. Images are taken from the camera and stored onto a computer.
9. ImageJ software 1.45 s [1] is loaded and the following steps are taken in order to analyse the images using ImageJ. Analysis of accuracy steps can be automated by using the record macros option in ImageJ.
  Image analysis and accuracy check (ImageJ)
  Image analysis steps
  Analysis steps describe the method for processing images by ImageJ freeware.
  a Open image – (file: open) – Fig. 2(a) is an example of an unanalysed image
  b Convert image to 16-bit – (image: type: 16-bit)
  c Subtract image background – (process: subtract background…) – set “Rolling ball radius” = “20.0 pixels” – tick “Light background” and “Preview” – Click “OK”
  d Adjust threshold – (image: adjust: threshold…) – set bottom bar between “65528” and “65532” whichever value gives the best cover for the image – click “Apply”
  e Separate overlaps – (process: binary: watershed)
  f Set measurements to analyze – (analyze: set measurements) – tick “Area”, “Standard deviation”, “Min & max gray value”, “Integrated density”, “Area fraction”, “Mean gray value”, “Perimeter” and “Median”
  g Carry out cell count – (analyze: analyze particles) – set “Size” = “100-Infinity”, “Circularity” = ”0.00–1.00” and “Show” = “Outlines” – tick “Display results”, “Summarize” and “Include holes” – click “OK” – Fig. 2(b) shows an example of the resultant generated image “Drawing of X.JPG” showing the cells that have been counted.
  h Save results – save “Results” and “Summary” as Excel worksheets. The summary file contains an ‘area fraction’ field. This is the percentage area that is covered by cells identified in the images. This value is used as a quantitative measure of cell confluence (see step 11 below).
    Accuracy check (ImageJ)
    Accuracy steps ensure that the count generated from the analysis steps is a good fit for the amount of cells in the original photograph. This is done by overlaying the generated image onto the original image to see if the black count outlines from the generated image match the cells seen in the original image. Images generated from the accuracy steps should result in an image where black outlines are inverted to red which outline the edges of cells from the original image. See Fig. 2(d).
  i Select generated image from analysis – click on image generated from step ‘g’ e.g., “Drawing of X.JPG”
  j Invert image – (image: lookup tables: invert LUT)
  k Change colour to red – (image: lookup tables: red)
  l Invert image again – (image: lookup tables: invert LUT)
  m Change both images to RBG colour (image: type: RBG colour) – Fig. 2(c) shows “Drawing of X.JPG” after accuracy steps ‘i–m’.
  n Merge images and see overlay (process: image calculator…) – set “Image1” = “Original image”, “Operation” = “Add” and “Image2” = “Generated image” – tick “Create new window” – click “OK”
  o Save image if accurate. Fig. 2(d) shows an example of an accurate overlay.
  p If the image is not accurate step ‘d’ (adjusting threshold) and step ‘g’ (“size” parameter) of the analysis steps can be adjusted until a good fit is obtained (i.e., each cell is labelled in red).
10. All analysis and accuracy steps are taken for the other photograph representing a flask (one from the top and one from the bottom is analysed for each flask).
11. Each area fraction (AF) output is stored in the summary file. The average of the two area fractions are calculated in order to give an AF which represents an approximate measure for surface area cover or confluence over the flask as a whole.
  a An average AF value of 30 or above represent a confluent T25 flask for both OVCAR8 and UPN251 cells. Fig. 3 shows pictures of OVCAR8 cells before and after analysis for newly seeded and confluent flasks.
  b The cut-off value for AF confluence is cell line dependant and therefore can be changed to suit different cell lines.

## Method validation

OVCAR8 and UPN251 cells were plated into eight T-25 flasks at a cell density of 2.6 × 10^4^ cells per flask. The flasks were left in an incubator for 24 h to allow cells to attach. After this time two of the flasks were removed and photographed, followed by AF output calculation. The two flasks were then trypsinised and cells counted using a haemocytometer. The remaining flasks were incubated and the same procedure was used over subsequent 24 h periods. AF output data was compared to cell count data using a Spearman correlation to see if the data sets correlated.

**Fig. 4:**
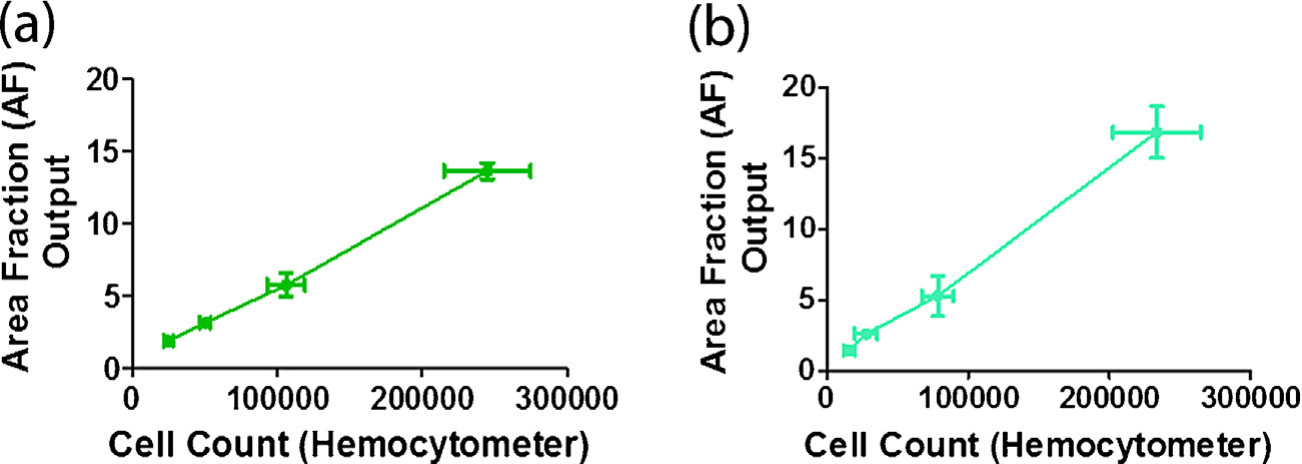
Correlation of AF method with cell counts using a haemocytometer. The average total cell count per T25 flask for each day of a four day growth curve is shown on the *x*-axis of each graph and the AF output average for the same T25 flask for each day is shown on the *y*-axis. For each graph error bars represent the standard deviation. (a) Shows a representative graph for OVCAR8. (b) Shows a representative graph for UPN251.

AF calculation was found to be highly correlated with cell count results using a haemocytometer. The average correlation co-efficient value obtained from a Spearman correlation test for *n* = 3 replicates was 0.99 ± 0.008 for OVCAR8 and 0.99 ± 0.01 for UPN251 with a *p*-value of 0.01 for both cell lines. See Fig. 4. This shows that AF calculation is a reliable measure for surface area cover of cells within a T25 flask and that this corresponds to cell count.

### Working example of using area fraction output calculation method: tracking recovery from drug administration

**Fig. 5:**
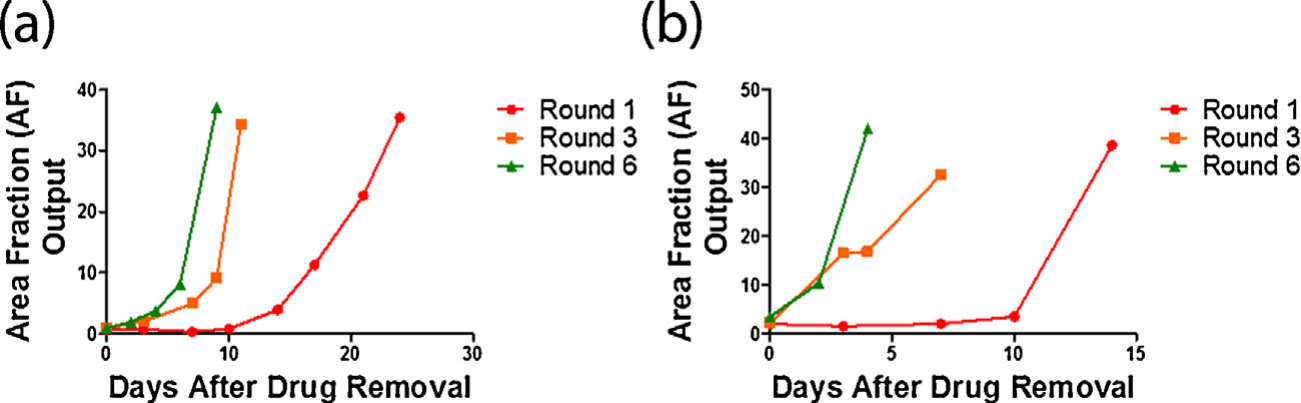
Tracking cell recovery using AF. Graphs show the recovery of cells over a period of days after carboplatin exposure. The cells received 3-day exposures to carboplatin every 4–5 weeks. Each dose of drug received, denotes a new round of drugging (Round 1–6). The *x*-axis shows the days after the drug has been removed from the cells and the *y*-axis gives the AF output at each time point measured. (a) OVCAR8 cells after exposure to 4 μg/ml carboplatin. (b) UPN251 cells after exposure to 2 μg/ml carboplatin.

One example where the application of the AF output method is useful is in the development of drug resistant cell models *in vitro*. AF output can be used to track cell recovery in an objective manner to see consistently when cells have recovered after drug exposure. Cells were plated into T25 flasks at a cell density of 2.6 × 10^4^ cells per flask and drugged with carboplatin (St. James’ Hospital Pharmacy, Dublin, Ireland) on day 2. The doses of carboplatin used were 2 μg/ml for UPN251 and 4 μg/ml for OVCAR8. These doses caused a similar level of death in the cell lines due to different intrinsic resistances to the drug. On day 5 drugged media was removed and replaced with fresh drug free media. Over subsequent days all T25 flasks were examined for confluence using the AF output method. Upon reaching confluence, cells were re-seeded into T75 flasks. Once all cells had recovered, the next round of drugging commenced following the same format as above (provided the cells were 4 weeks after drugging, otherwise drugging was delayed until this time). Fig. 5 shows the recovery of UPN251 and OVCAR8 ovarian cancer cells after carboplatin exposure (2 and 4 μg/ml, respectively) over a number of rounds of drugging during a selection strategy to produce carboplatin-resistant models. The doses of carboplatin that were used, were selected from testing a range of carboplatin doses on each cell line and selecting the dose which exhibited a large amount of cell death followed by a return to logarithmic growth (data not shown). Each cell line received 3-day exposures to carboplatin every 4–5 weeks, with each dose of drug received denoting a new round of drugging (Round 1–6). Generally, as the amount of rounds of drug administration increase the time taken for cells to recover decreases.

## Additional information

Many microscopy-based methods for measuring confluence use expensive equipment with specialised application software which is a good deal more expensive than the AF output method to implement. This may not be suitable for researchers on limited budgets. One example is the ‘cell screen’ which requires sample manipulation and use of an inverse microscope equipped with 10× objective and a back-illuminated charge-coupled device (CCD) camera which is operated by special application software [2]. Other examples use a 10× objective and QPm software in order to analyse bright-field images by creating phase maps [3], a microscope with an automated/motorised stage and an incubator attached controlled by the Metamorph^®^ image analysis software package [4] and digital holography using a Holomonitor™ M2 requiring automated software algorithms based on the Fresnel approximation [5].

Two recent studies have provided microscopy based methods of measuring adherent cell confluence which are quick, cheap, non-destructive and non-invasive [6], [7]. These studies measure cell confluence in 12-well plates and petri dishes. Neither of these studies measured cells directly in tissue-culture flasks as in our method. This novel aspect of our study makes it suitable for measuring cells directly in tissue culture flasks without disturbing growth.

Another recent publication provides and alternative method for analysing phase contrast microscopy images which is cheap, fast and accurate [8]. The software toolbox (PHANTAST) which bundles together different algorithms while providing a user friendly interface has been made freely available by the study.

Also classical membrane integrity assays are not suitable for the continued undisturbed growth of adherent cells in culture. Cell counting with a haemocytometer, trypan blue assay or automatic trypan blue method [9], lactate dehydrogenase (LDH) leakage [10] and florescent dyes (e.g., ethidium bromide, acradine orange and propidium iodide [11], [12] all call for invasive cell manipulations. In addition colourimetric assays such as crystal violet [13], MTT [14], Resazurin [15] and BrDU [16] are also unsuitable for measuring cell confluence in an undisturbed manner as they are either destructive or can only be used as end point assays. Our method of AF output has been optimised for epithelial cells using the outlined settings and conditions above. By modifying the point of focus when capturing images, adjusting the threshold settings (step (d) image analysis) or “size” parameter (step (g) image analysis), other cell types could be optimised to use this cheap and accurate method of measuring confluence. Also the cut-off value for AF output confluency measure can be adjusted for cells of different morphologies. For example, for cells with a smaller nuclei and larger cell body, the AF output cut-off value could be lowered to reflect this.

## References

[1] Schneider C.A., Rasband W.S., Eliceiri K.W. (2012). NIH Image to ImageJ: 25 years of image analysis. Nat. Methods.

[2] Brinkmann M., Lutkemeyer D., Gudermann F., Lehmann J. (2002). New technologies for automated cell counting based on optical image analysis; The Cellscreen'. Cytotechnology.

[3] Curl C.L., Harris T., Harris P.J., Allman B.E., Bellair C.J., Stewart A.G., Delbridge L.M. (2004). Quantitative phase microscopy: a new tool for measurement of cell culture growth and confluency in situ. Pflugers Archiv.: Eur. J. Physiol..

[4] Juneau P.M., Garnier A., Duchesne C. (2013). Selection and tuning of a fast and simple phase-contrast microscopy image segmentation algorithm for measuring myoblast growth kinetics in an automated manner. Microsc. Microanal..

[5] Molder A., Sebesta M., Gustafsson M., Gisselson L., Wingren A.G., Alm K. (2008). Non-invasive, label-free cell counting and quantitative analysis of adherent cells using digital holography. J. Microsc..

[6] Drey L.L., Graber M.C., Bieschke J. (2013). Counting unstained, confluent cells by modified bright-field microscopy. Biotechniques.

[7] Topman G., Sharabani-Yosef O., Gefen A. (2011). A method for quick, low-cost automated confluency measurements. Microsc. Microanal..

[8] Jaccard N., Griffin L.D., Keser A., Macown R.J., Super A., Veraitch F.S., Szita N. (2014). Automated method for the rapid and precise estimation of adherent cell culture characteristics from phase contrast microscopy images. Biotechnol. Bioeng..

[9] Louis K.S., Siegel A.C. (2011). Cell viability analysis using trypan blue: manual and automated methods. Methods Mol. Biol. (Clifton N.J.).

[10] Allen M., Millett P., Dawes E., Rushton N. (1994). Lactate dehydrogenase activity as a rapid and sensitive test for the quantification of cell numbers in vitro. Clin. Mater..

[11] Jones K.H., Senft J.A. (1985). An improved method to determine cell viability by simultaneous staining with fluorescein diacetate-propidium iodide. J. Histochem. Cytochem..

[12] Ribble D., Goldstein N.B., Norris D.A., Shellman Y.G. (2005). A simple technique for quantifying apoptosis in 96-well plates. BMC Biotechnol..

[13] Gillies R.J., Didier N., Denton M. (1986). Determination of cell number in monolayer cultures. Anal. Biochem..

[14] Mosmann T. (1983). Rapid colorimetric assay for cellular growth and survival: application to proliferation and cytotoxicity assays. J. Immunol. Methods.

[15] Anoopkumar-Dukie S., Carey J.B., Conere T., O’Sullivan E., van Pelt F.N., Allshire A. (2005). Resazurin assay of radiation response in cultured cells. Br. J. Radiol..

[16] Muir D., Varon S., Manthorpe M. (1990). An enzyme-linked immunosorbent assay for bromodeoxyuridine incorporation using fixed microcultures. Anal. Biochem..

